# Care Model Design for E-Health: Integration of Point-of-Care Testing at Dutch General Practices

**DOI:** 10.3390/ijerph15010004

**Published:** 2017-12-21

**Authors:** Bart Verhees, Kees van Kuijk, Lianne Simonse

**Affiliations:** Department Product Innovation Management, Faculty Industrial Design Engineering, Delft University of Technology, Landbergstraat 15, 2628 CE Delft, The Netherlands; B.F.Verhees@student.tudelft.nl (B.V.); K.J.vanKuijk@student.tudelft.nl (K.v.K.)

**Keywords:** point-of-care testing, modelling business models, visual modelling, general practice, diagnostic centre, Dutch healthcare system

## Abstract

Point-of-care testing (POCT)—laboratory tests performed with new mobile devices and online technologies outside of the central laboratory—is rapidly outpacing the traditional laboratory test market, growing at a rate of 12 to 15% each year. POCT impacts the diagnostic process of care providers by yielding high efficiency benefits in terms of turnaround time and related quality improvements in the reduction of errors. However, the implementation of this disruptive eHealth technology requires the integration and transformation of diagnostic services across the boundaries of healthcare organizations. Research has revealed both advantages and barriers of POCT implementations, yet to date, there is no business model for the integration of POCT within general practice. The aim of this article is to contribute with a design for a care model that enables the integration of POCT in primary healthcare. In this research, we used a design modelling toolkit for data collection at five general practices. Through an iterative design process, we modelled the actors and value transactions, and designed an optimized care model for the dynamic integration of POCTs into the GP’s network of care delivery. The care model design will have a direct bearing on improving the integration of POCT through the connectivity and norm guidelines between the general practice, the POC technology, and the diagnostic centre.

## 1. Introduction

The emergence and disruption caused by point-of-care testing (POCT) technologies has been observed and documented since the 1980s [1]. POCT or near-patient testing basically refers to laboratory testing with new mobile devices and online services outside of the central laboratory, in the context of bedside care or self-care. Instead of specialized laboratory personnel, such diagnostic eHealth devices typically target new types of users who have a more general or even no diagnostic background. The devices may incorporate highly advanced molecular technologies, but can also involve less complex regular tests that have been made available to the general public with mobile internet technologies [2,3]. Research showed that the use of the POCT devices implicates a shift in diagnostic practice across organizations. First, it brought laboratory testing closer to the hospital bedside [4], then it decentralized diagnostic testing by taking it out of the controlled environment of hospitals to general practitioners (GP) and nursing homes [5], and then eventually, introduced it into the homes of people as well, creating the self-testing market [4]. Already in 2002, a study by Bissell and Sanfilippo [4] showed that POCT devices and supplies accounted for a fourth of the total market for clinical laboratory in vitro diagnostic products. Nowadays, the POCT market is outpacing the traditional laboratory testing market, growing at a rate of 12 to 15% each year [1]. 

Moreover, an increasing number of new mobile devices and online services for POCT has an important role in the lives of patients, and for this reason, they demand to have greater say in the diagnosis and treatment process. The health professional and the patient collaborate in obtaining and analyzing the information that affects the diagnosis—this is a process called co-diagnosis [6,7,8].

In this research, we focused on the shift of laboratory tests to primary care, with particular attention on the integration of these mobile devices and eHealth solutions of POCT at general practices.

### 1.1. Impact Effects

The GP is the first health professional in line that can make use of POCT in the official healthcare system, and thus plays an important role in the decision-making process and the economic outcomes after the consultation. Economic outcomes include fewer unnecessary hospital admissions, and better optimized or less inappropriate use of drugs [3]. Further research concerning the impact effects has shown that POCT yields high efficiency benefits, such as a decrease in both turnaround time [9], and the time between the acquisition of the sample and its analysis [5]. Moreover, POCT shortens the total time of the diagnosis process for caregivers because the instant decision-making process is not delayed by transport or external laboratory activities [3,10,11]. In addition, quality benefits in the form of fewer analytical errors are reported, evidencing more effective healthcare delivery [12]. Apart from research at an intensive care unit, that showed a direct impact relation between the decrease in TAT and saving lives [12], the effectiveness of improved medical outcome of patients has not been fully demonstrated [1]. The most successful example of POCT for patients is glucose testing and insulin pumps, that provide chronic diabetes patients with an independent lifestyle [1]. POCT can have the potential to empower patients in their own care pathway [13], because it provides a direct test result, and enables an immediate discussion about the treatment. Patients judge the effectiveness of doctor–patient communication using the doctor’s “bedside manner” as a major indicator [7]. This empowerment has already started with the self-diagnosis practices, in which patients use information they find online [6]. In addition, POCT and empowerment can thereby lead to improved consent in disease management [14], and according to Gialamas (2009), patient satisfaction increases as well. 

### 1.2. Advantages and Barriers of Transformations

In reviewing the literature on POCT technologies, we came across several specific advantages and barriers. For instance, POCT suits large-scale disease-control programs in third world countries, as it provides instant access and large-scale availability over widespread areas [11,15]. The indirect cost advantages of POCT relate to the smaller size of (capital-intensive) measuring equipment, and the reduced amount of specialized knowledge needed by the caregiver to perform a reliable diagnosis [5]. However, cost barriers have also been reported. At general practices and nursing homes, POCT implementation requires a relatively high starting investment, followed by additional fixed running costs per test [16]. Other barriers have to do with the necessity of training staff and maintaining the equipment [12,17]. This extra work, on top of the already high workload that is commonly experienced in primary care, is the highest concern [17]. The paradox is that on the other side of the health system, in secondary and tertiary care, there is a barrier of resistance with respect to the decentralization of the diagnostic work activities. Research revealed that laboratory professionals at hospitals are highly critical of the trustworthiness of testing activities outside labs. They consider the uncontrolled test settings of POCT a barrier [17,18], and argue that it is not only the test itself that is important, but also the follow-up within the health system; only if the POCT diagnosis results lead to immediate therapeutic decisions, do they consider POCT useful [5]. 

### 1.3. Integrated Care Challenge with Connectivity and eHealth

Overall, the success and impact of POCT depends on its integration into the health system across organizations. According to Pai [17], this can be done by creating POCT programs that demonstrate how to implement the POCT tests that lead to public health actions, based on the test results [10,11]. As a pre-condition for successful integration in the health system, this requires overcoming operational and administrative challenges. The current support of administrative services across organization is frail, in particular, with respect to product regulation, supply chain, human resources and training, quality assurance, and equipment maintenance [15]. Connecting organization through internet technologies poses a key trigger for integrating POCT: “Connectivity is the key to managing testing that takes place at many sites, including aspects such as quality control (QC), identification of testing personnel, and post-analytical transfer of results to the patient’s electronic medical record” [1]. Such connectivity issues are common to many eHealth devices and online services, in the sense of eHealth, defined by the WHO as “the cost effective and secure use of information and communications technologies in support of health and health related fields” [19]. However, with IT, eHealth, and mobile and wireless connectivity widely available and proven for some POCT equipment [1,15], there is a lack of actual value creation. For the integration of a disruptive innovation, like POCT, within the health system, care models are missing. For its adoption by healthcare providers, these providers need to revise their strategy, and therefore, their business model [20,21]. We ground our research on Amit and Zott who developed a model of the sources of value creation: “A business model depicts the content, structure, and governance of transactions designed so as to create value through the exploitation of business opportunities” [22]. They proposed that a firm’s business model is an important locus of innovation, and a crucial source of value creation for the firm and its suppliers, partners, and customers. In order to create a business model for GPs that enables them to keep up with the ongoing development of POCT, and to deal with the dynamics and ongoing development of POCT, we take as research objective to establish the most effective means whereby all actors can integrate POCT within the general practice; we define this as the optimized business model. Therefore, as a research question, we formulated: What is the business model for point-of-care testing at a general practice (1), and how should an optimized business model be designed to integrate point-of-care testing within the general practice (2)?

## 2. Materials and Methods 

**Figure 1:**
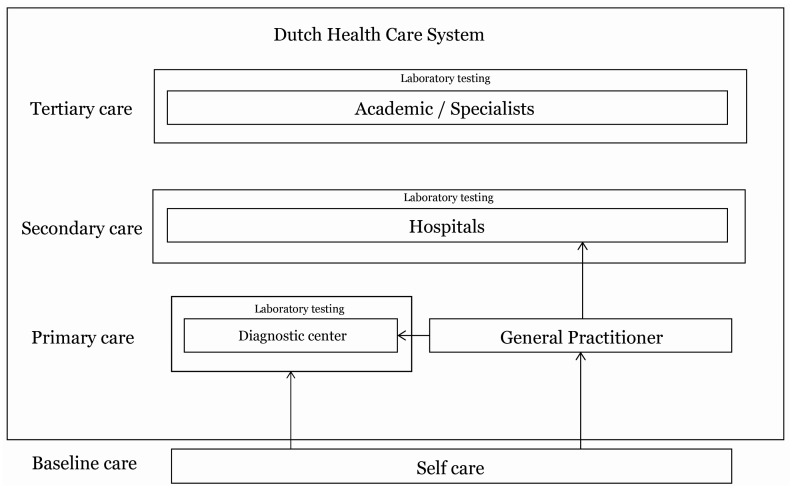
Overview of the Dutch healthcare system.

The healthcare system in The Netherlands, in which the three most prominent health care providers are the GPs, hospitals, and nursing homes [18] (see the overview in Figure 1), is the context of this research project. GPs have an important role as gatekeeper in the Dutch healthcare system. They provide the first professional contact for the patient in the healthcare system [15]. In order to understand the value and meaning of POCT from the perspective of GPs [23,24] we employed a method of qualitative research with scientific rigor. For retrieving rich data on the challenge of integrated care for the eHealth technology of POCT and the care model of value creation, we used a design research approach for business model design [25,26]. To this end, it was necessary to determine which parameters determine a business model in a health system, and which data would suit the design of a care model [24]. Therefore, we first defined the parameters of the business models [25]. Second, we crafted a design model toolkit by performing a preliminary research with a general practitioner. Third, we conducted the design research with the materials of the design model toolkit to support our interviews, to retrieve credible, relevant, and transferable data to answer our research question.

### 2.1. Business Model Parameters

For modelling care models, we follow Simon who stated that “*modelling is a principal tool for studying the behaviour of complex systems” and “to manage this complexity we must separate what is essential from what is dispensable in order to capture in our models a simplified picture of reality which, nevertheless, will allow us to make the inferences that are important to our goals.”* [27]*.* We started from the construct definition of a business model design by Amit and Zott: “*A business model depicts the content, structure, and governance of transactions designed so as to create value through the exploitation of business opportunities”* [22]. “Depicts” refers to the model as a visible artefact, a visible representation of the business model. “Content of transaction” refers to the information that is being exchanged and the IT, the connectivity that is required to enable these exchanges. “Structure of transaction” refers to the revenue structure of the model in which multiple actors are participating and exchanging money. “Governance of transactions” refers to the ways in which flows of information, money, and value creation are controlled by the relevant actors and the legal form of the organization [25]. Based on this widely adopted and most cited business model definition, we defined as design parameters: (a) the information and connectivity, (b) the revenue, (c) the value, (d) the transactions, and (e) actors’ structure, and (f) the governance. 

### 2.2. Preliminary Research—Crafting the Design Model Toolkit

To model the value, revenue, and connectivity transactions of the care delivery network of a general practice, we conducted preliminary research to define the key actors within the operations of a general practice that directly influences the integration of POC devices and online services [28]. We determined an actor as an entity that has a direct impact on the transactions influencing the integration of POCT within the general practice [9]. The initial actors were captured from the literature [29], and presented to a GP who participated in this preliminary research as an expert. This revealed that three main actors, the health insurer, the Dutch College of General Practitioners (NHG) and hospitals in secondary care directly influence transactions within a general practice from the outside in, and therefore, have an impact on the integration of POCT. In the preliminary research, the GP stated: “The aspects that are the most relevant to our general practice are revenue, value transactions and information technology and management; they are therefore essential for POCT integration.” [28]. 

**Figure 2:**
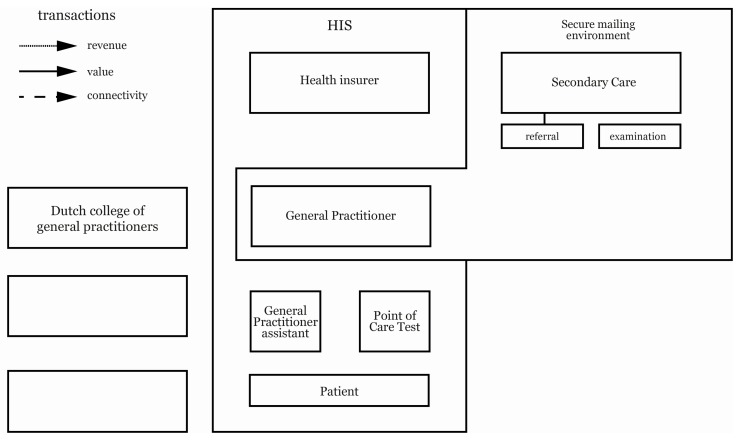
The design model toolkit. HIS: a general practice information system.

The patient and the general practice assistant—the actual user of the POC devices—also have a direct influence on POC product integration [28]. The POC device itself can create value and revenue for the GP by yielding additional information for diagnosis and treatment opportunities. This also depends on the demographic area of a GP, and the support for the usage of a particular POCT. All the actors, except NHG, are connected by information systems that have a direct influence on connectivity transactions within the business model. These systems are, therefore, key to implementing POC products [1]. To start, every general practice has a general practice information system (HIS) [30]. This is an internal system that is connected to all internal actors, and HIS is also used to submit declarations to the health insurer. In addition to the HIS, GPs use a secured mail environment, such as Zorgdomein or Zorgmail, to communicate with secondary care and refer patients in a secure environment [31]. These two IT systems are therefore included in the design model toolkit, see Figure 2. 

The design modelling toolkit is crafted based on the visual business modelling toolkit that depicts the transactions between actors in a network [26]. The toolkit aims to visually capture connections, and to monitor and evaluate situations involving many stakeholders who influence potential outcomes. Our design modelling toolkit consists of all the key actors, as explored in the preliminary research, and we used our interview guide to map the different transactions between the actors. The toolkit consists of a large sheet with cards for all of the actors involved. Revenue, value and connectivity transactions were physically mapped on these cards according to the insights of the general practice owner. Blank actor cards were also included to allow the interviewee to introduce new actors into the business model. We used different symbols on the arrows to depict the specific transactions during the interview: revenue, value, or connectivity. To improve the transferability and comprehensibility of the model, we used contextual definitions to indicate the content of these transactions, because presenting symbols in isolation limits comprehensibility [32]. Together with the interview guide, the toolkit structured the interview, and created consistency and reliability in the data collection across interviews. 

### 2.3. Design Research

In the design research, semi-structured interviews were conducted to map the POCT business model of general practices. A total of seven interviews was conducted: five with general practice owners, and two with innovation managers at a diagnostic center. The main characteristics of these practices can be found in Table 1. We focused our sample, first, on one particular actor, the general practitioner, to capture the in-depth findings from their particular view. However, in the fourth and fifth interview, a saturation on data collection was reached. Although we had access to more interviewees, no significantly new data was obtained. Therefore, we turned to a second actor that emerged as highly influential, the diagnostic center, and interviewed two innovation managers, and focused on the interactions between the diagnostic center and the general practice. 

An interview guide was constructed to ensure that the same topics of inquiry would be pursued, while enabling the researchers to examine transaction topics of interest with each of the interviewed GPs in breadth and depth [33]. 

Since we focused on the business modelling part of POCT, we compared well-integrated POC products with discarded POC products in the general practice. We defined a well-integrated POC product as a product that is included in the operations of the general practice, and a discarded POC product as a product that was first included in the general practice, but was discarded due to transaction failure. By comparing these two different integration business modelling outcomes, we obtained data to construct an optimized business model for POCT integration. The visual business modelling toolkit, together with the interview guide, structured different POCT business models of the general practice, and created consistency across the interviews with different general practitioners. 

### 2.4. Data Analysis

To ensure the validity of our findings, we accommodated triangulation. Three types of qualitative data were analyzed: business models, audio recordings, and documentation. After each interview, sheets with multiple business models were digitally reconstructed in Illustrator, to establish order, clarity, and completeness. The eight models that were generated were analyzed at the actor and transaction level. First, the actors within the models were compared to determine whether new actors had emerged compared to the business modelling toolkit. Secondly, the transactions between the actors were comparatively analyzed, to determine which transactions arise between the actors in the different models. The transactions were separately compared at the value, revenue or connectivity level, and their frequencies were established. At last, a table was created of the visualized business models, including the actors and transactions, thereby providing a clear overview of all the transactions and their frequencies. 

The interviewees mentioned a variety of qualitative information that could not be included in the established models, but which has an influence on POCT integration; such value information is listed in a separate table. We retrieved this data by listening to the audio recordings and comparing them with the documentation we made during the interviews. For the credibility and confirmability of our qualitative findings, we selected only the data that occurred in at least three of the five interviews. Findings that had no influence on the integration of POCT were excluded.

### 2.5. Designing the Care Model

**Figure 3:**
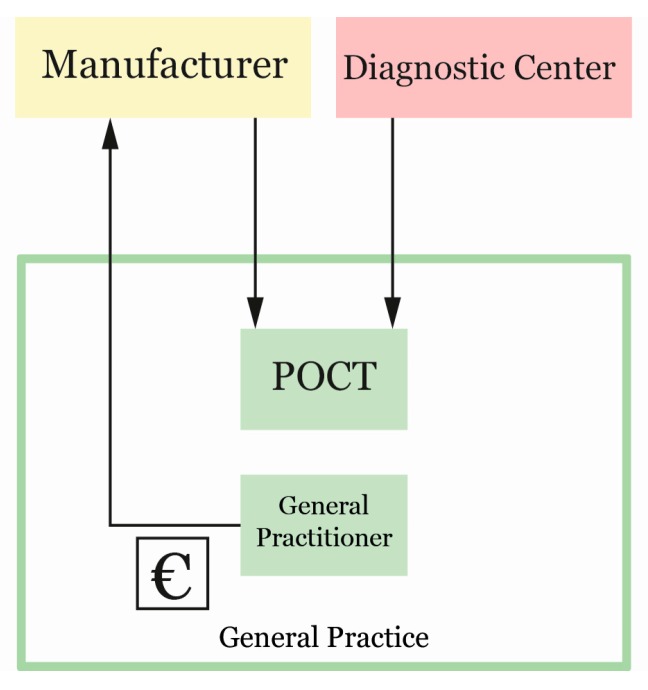
Business options for obtaining point-of-care testing (POCT).

The table that we established from the business models formed the input for answering the first part of our research question; (1) What is the business model for point-of-care testing at a general practice. We used our modelling and design skills to create two different existing point-of-care business models for the general practice. The design process was an iterative process consisting of the analysis, synthesis, and evaluation of this table. A new key actor—the diagnostic center—emerged from the results of the interviews with the GPs, as a direct source of obtaining POC technology (see Figure 3). To obtain a validated answer to the second part of our research question, an in-depth inquiry was conducted with this newly obtained actor that has a key influence on POCT integration. Two interviews were conducted at the Saltro diagnostic center in Utrecht, one of the largest diagnostic centers in The Netherlands. Saltro has an innovation department within the center that enables the integration of POCT at the general practice level. We interviewed, first, a POCT coordinator and secondly, a POCT specialist with a GP background. We used the design modelling toolkit with the POCT specialist to map the transactions between the actors involved in POCT integration from the perspective of the diagnostic center. The model that we generated with the POCT coordinator focused only on the coordination of information and value between the diagnostic center and the GP, instead of the total business of the diagnostic center. This split in modelling was used to obtain the most detailed information on all the transactions between the actors at a single diagnostic center. The information gathered during all interviews with the general practitioners, the diagnostic center, and the business models created for the general practice and the diagnostic center formed the input of the optimized business model to integrate POCT within the general practice.

## 3. Results

Table 2 presents the different transactions that currently take place between the GP and the different actors. These transactions are divided into value, revenue, and connectivity transactions, as explained in the methodology. The transactions are extracted from the eight business models established during the research. We counted the occurrences within the models; if a transaction is referred to more frequently, it has more value within the business model of the general practice. The overview of Table 2 we took as a baseline for the business model design of POCT integration in the general practice. 

### 3.1. Findings from GPs That Influence Integration of POCT

In addition to the findings of transactions, the research revealed that general practice owners mainly focus on providing good care for their patients, and are not actively seeking new ways to create extra revenue for the practice. One GP said: *“My salary is good, I care about my patients and I focus on their treatment. Our practice is not looking for opportunities to earn extra money from them.”* These findings, that possess in-depth qualitative information that influences POCT integration within the general practice, are listed in Table 3.

Table 4 shows problems that occur in practice with the integration of POCT. These findings reveal that the general practitioners do not consider themselves as an incubator of POC products; and have a risk-averse attitude towards new diagnosis products, demanding a complete validation of a new POC product before using it for the diagnosis and treatment of their patients. One GP said: “I am not going to burn my hands”. Most of the problems in Table 4 relate to the product itself. Many GPs told us: “A too large grey interoperation area is not useful for these products, because then it adds no value to me or my patient.” The test outcome, depending on the technology, needs to be reliable, and the product cannot be too complex for the GP’s assistant to use, considering lack of time or education level. We concluded, based on this information, that the first requirements for POCT integration concern the reliability and usability of the product. In addition, the test result must yield a decisive answer or additional value for the diagnosis of the patient. 

### 3.2. Point-of-Care Business Models of a General Practice (1)

The results shown in Table 2, Table 3 and Table 4 provided the basis for answering the first part of our research question. Next, we generated visual models to design a business model of POCT within the general practice. We transferred and modelled the complex information into a clearer model that gives an overview of the transactions between the actors. We classified the transactions as a value, revenue, and connectivity transaction, according to the statements of Table 3, and created a generic model, applicable to the dynamics of multiple POCT products. As a result, two business models were created with the same transactions between the actors, except for the obtaining part of a POCT product for the practice. 

**Figure 4:**
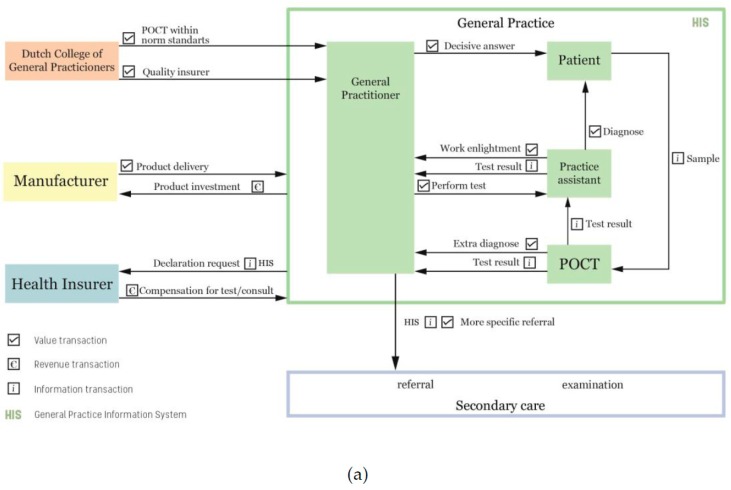

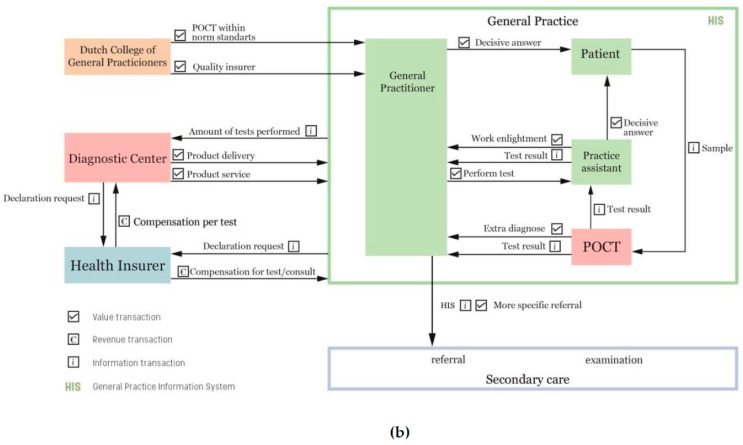
(**a**) Point-of-care business model with Diagnostic Centre; (**b**) Point-of-care business model with manufacturer.

Figure 4a presents the current business model with the diagnostic center as a supplier. The POC product interacts with the actors within the general practice and with the diagnostic center, since they supply the practice with a POCT service. A diagnostic center is originally established by GPs to improve the quality of the health care. Nowadays, diagnostic centers support the GPs by providing diagnostics for patient diagnosis. Firstly, they do so by carrying out laboratory research for GPs and individuals. Secondly, the centers serve as innovation labs for POCT. They test the reliability of POCT products by using their highly reliable laboratory test results to benchmark the test results of POC products. Diagnostic centers offer widely validated POC products to general practices in a form of a pilot or as a complete service. In both situations, the diagnostic center is a supplier of POCT equipment, materials, and service to the general practice, including maintenance, calibration, retraining of GP assistants, and creating usage awareness. Diagnostic centers are non-profit organizations whose mission is to serve as POCT quality controllers for the GPs. Figure 4b shows the business model when a GP invests in the product and buys it from a medical manufacturer. These business models present the content, structure, and governance of transactions that provide the general practitioner and the patient with value through a business opportunity for the general practice. 

### 3.3. An Optimized Business Model to Integrate Point-of-Care Testing within the General Practice (2)

**Figure 5:**
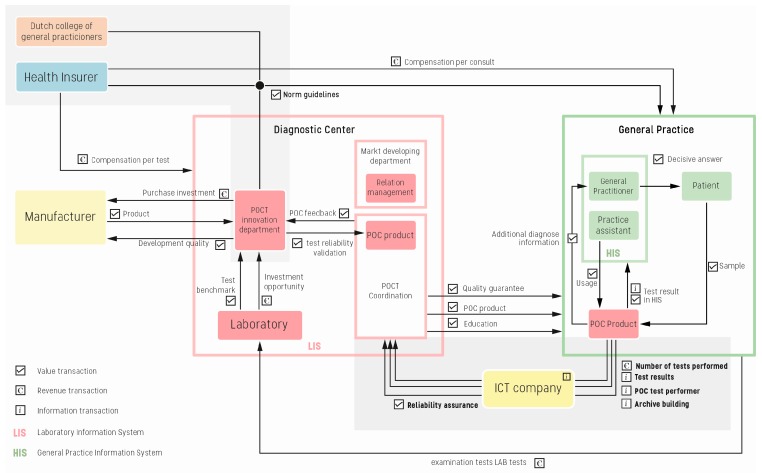
Optimized business model for point of care integration within the general practitioners’ organization.

To rephrase, the objective of this research was to create the optimal business model that integrates POCT within the general practice. As stated in the introduction section, “optimal” is defined as making the most effective use of the actors, and this means creating additional value and/or revenue transactions for the participating actors. By adjusting the value, revenue, and connectivity transactions between the actors, we designed a more optimal business model for these actors. In addition to the information gathered during the interviews with the general practitioners, we interviewed professionals at the diagnostic center, and created a more detailed and optimal business model for the network of general practices and the diagnostic center. Figure 5 presents the optimized business model that answers the second part of our research question; how should an optimized business model be designed to integrate point-of-care testing within the general practice. 

Compared with the current business models of the general practice, the focus of the optimized model shifts from the interaction within the general practice towards the interaction between the diagnostic center and the general practice. The research at the diagnostic center resulted in a detailed description of the actors and transactions that occur within the diagnostic center. Therefore, these transactions are also visualized in the optimized model. Overall, this optimal model is based on the two POCT business models of the general practice, shown in Figure 4a,b, and the findings, summarized in Table 3 and Table 4, that determine the POCT integration, especially with respect to the reliability and usability of the POC product. Since the diagnostic center has influence on these requirements, the business model of the general practice transforms into a more elaborated model that includes more details of the diagnostic center. The diagnostic center is a key player in the optimal integration of POCT within the general practice.

In comparison with the current business models, the optimized model decreases the number of actions related to POCT usage. This is realized by connecting the POC product to the HIS (General Practitioners’ Information System, the internal IT system every practice uses) and with a direct connection to the diagnostic center. This eliminated the manual reporting action of the GP’s assistant, and the GP to the HIS and to the diagnostic center, which means that the results are not compromised by human error. The impetus for the implementation of this connection comes from the diagnostic center; it has the most value to gain from connecting the product to its laboratory information system (LIS). With this connection to the LIS, laboratory information system, the diagnostic center can check the quality and reliability of the POC technology, and declare the test with the health insurer. Diagnostic centers need connectivity between the POC product and LIS; without knowledge of the occurrence of human or technological errors, they cannot assure quality control. According to the POCT coordinator, this is the biggest problem facing medical manufacturers that supply POC products to practices, as they do not provide this quality control service: “We need this quality control to know what is happening with our products in these practices.” This quality and reliability check directly solves the two problematic value transactions, see Table 4, between the GP and the POC product. It ensures that a quality check is performed by a professional organization specialized in laboratory testing. Therefore, in order to connect the diagnostic center with the general practices, they need secure information and communication technology to support POCT as an eHealth service. The POCT specialist with a GP background said: “We need an external partner to fix this ICT problem, however this will cost money and effort. Yet the general practitioners do not directly benefit from the connection to the LIS, they just want to use the product for its direct value to the patient.” Besides adding connectivity to optimize the coordination of POC products within the general practice, the involved actors need to compose new guidelines for specific POC products in the general practice. Currently, guidelines are created without the involvement of health insurers; however, the health insurer needs to participate in this process, because it is the party that compensates the users of POC products.

## 4. Discussion

In this study, we investigated the business models of POC products at the general practice. Our models depict the transactions that occur between the actors that are involved in the integration of POC products within the general practice. The designed optimized model of POCT within the general practices visualizes the optimal value, revenue, and connectivity transactions, and the critical phases for integration of POCT in the general practice. Its design is crucial for the integration of POC technology into the eHealth system. It identifies the necessary transactions that need to take place, (1) norm guidelines, (2) connectivity [1], and (3) revenue model [11], and the actors that need to be involved. This model overcomes the barriers stated in the literature [1,3,5,10,11,12]. It proposes the diagnostic center as the gatekeeper of POCT within the Dutch health care system. It is the first study to present a visual depiction of all the actors involved from the diagnostic center and general practices, and therefore, provides a detailed overview of all the insights in the integration process of POCT. Our design background contributed to the research—we harnessed our skills to create the modelling toolkit to generate visualized models that could be validated directly by the interviewee. The visualized models are highly transferable to all the actors, due to their structured presentation, in which words are used in combination with arrows and boxes; therefore, the information displayed is easily understood by everyone. In addition, our way of care modelling is different from other business models, because actors gain not only an overview of the context, but also a detailed description of the transactions from a low-level perspective [34]. We add to the theory of modelling business models [25], in that the value transaction mapping tool is only readable by all actors when descriptions of transactions are given in the final model. 

We consider this new business model suitable for all actors involved, to identify their role in the integration of POCT. Thanks to the detailed overview that it provides, this model is especially suited for the diagnostic center and the general practice. It helps the diagnostic center to demonstrate the importance of connectivity to general practitioners, and shows the other actors the importance of the diagnostic center in the integration process of POCT, due to the help the center provides in the form of necessary supporting services [15]. This model also mitigates any concerns that laboratory professionals and hospitals might have about POCT [18]. 

### 4.1. Limitations and Further Research

The semi-structured interviews, together with the design modelling toolkit, have proven to reveal in-depth insights into the object of analysis, yet there are limitations. A limitation of using an interview guide is that important and salient topics may be inadvertently omitted. Interviewer flexibility in sequencing wording questions can result in substantially different responses from different interviewees, thus reducing the comparability of the responses [33]. However, we used a toolkit during the interviews to process the obtained information immediately into a business model, enabling us to validate the business models on the spot with the interviewee. 

Another limitation of our research is the fact that we only included two actors, the GP and diagnostic center, in great detail in our research. The model could be improved by obtaining insights from other actors; however, for the purpose of POCT integration within the general practice, the optimized business model provides an overview and insights for all actors on how to function in this model. The optimizations incorporated into the POCT business models are derived from the expertise of both the diagnostic center and GPs; the diagnostic center, in particular, provided valuable and realistic insights into optimizations to improve the current POCT business models. When this optimized model is implemented in actual practice, iterations could be made on the model based on further research. This paper focused on the integration of POCT in the general practice. It should not be forgotten that this has an impact on the Dutch healthcare system as a whole. Mainly, it will influence interaction between the general practice, laboratory and health insurer, and therefore, future research is required to study the consequences. It seems that the health insurer benefits least from the current model, as the insurer needs to pay for the tests performed at the general practice and the diagnostic center. Further research is necessary to investigate the potential contribution of POCT to the decline in healthcare costs in total. Last but not least, the results of this paper are not directly transferable to other healthcare systems in other countries. Within The Netherlands, the diagnostic center is the innovator in POC technology. This innovator role is necessary to control the quality and accuracy of the technology. In other countries, such innovator roles need to be identified to establish a fertile situation for POC technology.

### 4.2. Professional Implications

In Figure 5, we presented the optimized business model for integration of point-of-care testing for the general practice. For the professionals involved in the integration of POCT, it is of interest to have an overview of the actors involved, and to see what kinds of transactions occur between them. The model creates awareness of what is necessary to integrate POCT. With this awareness, the general practitioner can play a more active role in creating norm guidelines and connectivity. This will have a direct bearing on improving the integration of POCT. The diagnostic center can use this model as a tool to demonstrate to general practitioners the necessity of working together with a diagnostic center, and of establishing a connectivity bridge between them. 

## 5. Conclusions

This paper defines the optimized business model of point-of-care testing of a general practice. The design of the model is an optimization of the current business models of point-of-care products at a general practice. The actors involved within the business model are (1) NHG, (2) health insurer, (3) diagnostic center, (4) general practice, (5) ICT company, (6) POCT product, and (7) manufacturer. The optimized transactions between them are creating norm guidelines and connectivity between the general practice, the POC product, and the diagnostic center. The two modelled business models (Figure 4a,b) of point-of-care testing in the general practice, in combination with the designed optimized model (Figure 5), answer the research question of this research.

## Figures and Tables

**Table 1:** Sample of interviewees.

Practice No.	Geographic Information	No. Patients in Practice	Supporting Hours	Demographic Information Patients	Specialty’s Practice	No. of GP’s in Practice
1	Rural	2900	65 h pharmacy assistant 66 h practice assistant 18 h practice support (chronicle diseases)	Aged population	Heart and vascular disease Intern pharmacy	2
2	Urban	8400	80 h practice support GGZ support 15 employees	Normal	Palliative	4
3	Urban		9 employees	Aged population	Heart and vascular disease	4
4	Urban	3000	20 h practice support (chronicle diseases) 1 GP in training 38 h practice support	Low multicultural rate	none	2
5	Urban	4670	24 h practice support 13 h GGZ support 80 h practice assistant		Palliative	3
6	Urban				Diagnostic Center	
7	Urban				Diagnostic Center	

GP: general practitioners.

**Table 2:** Analysis of the eight business models.

Actor View From	Actor to	Transaction		
		Value	Revenue	Connectivity
**GP**		-	-	-
...	Dutch College of General Practitioners (NHG)	∎∎∎∎ Quality guarantee of the treatment, reinsurance for the general practitioner (GP)		∎∎∎∎∎ NHG provides GP with Norm standards through (Website)
	Health Insurer	∎∎ Negotiating compensation rates	∎∎∎∎∎∎∎ Compensation consult on bank account ∎∎∎ Compensation test on bank account	∎∎∎ Consult compensation request in the General Practitioners Information System (HIS) ∎∎∎ Double consult compensation request (HIS) ∎∎∎ Test compensation request (HIS)
	Secondary care	∎∎∎∎ Specific referral ∎ Diagnosis from specialist ∎ Less referrals		∎ Sample photo ∎ to specialist ( *Zorgdomein*) Diagnose from specialist (*Zorgdomein*)
	Diagnostic center	∎∎∎∎ Delivers product and service to GP ∎∎ Acquisition of new POC products ∎∎∎∎ Quality insurer and calibration of POCT equipment		∎∎∎∎ Validation test result ( *Zorgmail*, *Zorgdomein*) ∎∎∎∎ Number of tests performed (*Zorgmail*, *Zorgdomein*)
	GP assistant	∎ Referral to GP when complex test result ∎ Create extra appointment to perform test		∎∎∎ Test result to GP from assistant (paper, verbal, HIS) ∎∎∎∎∎ Test to assistant (paper, verbal)
	POCT	∎∎∎∎∎∎ Extra diagnosis information, additional argument ∎ Creates sample for testing	∎ Product investment	
	Patient	∎∎∎∎∎∎∎∎ Complementary information and evidence for diagnosis to the patient ∎ Same day diagnosis from the specialist	∎∎∎∎ Test result to GP (paper) ∎ Test result to patient (telephone)	
**GP assistant**				
	Health Insurer			∎ Consult compensation request (HIS)
	POCT	∎∎∎∎∎∎∎ Perform tests for additional diagnosis information		Test result information (paper, visual)
	Patient	∎∎∎∎∎∎∎ Perform tests for direct diagnosis ∎ Patient brings sample for testing		∎∎∎∎∎∎ Test result to patient (paper, verbal)
**Health Insurer**				
	Secondary care		∎ Compensation for teledermatology diagnosis on bank account	∎ Test compensation request
	Diagnostic Center		∎∎∎∎ Compensation per test on bank account	∎∎∎∎ Test compensation request

GP: general practitioners. POC: point-of-care, POCT: point-of-care testing.

**Table 3:** Influence factors from GPs on POCT integration.

Number of GP’s	Findings from GP’s That Influence Integration of POC Products
∎∎∎∎∎	A GP has his own preferential areas to work in
∎∎∎∎	A GP has his specific work skills, subjective knowledge
∎∎∎∎∎	When a GP purchases any new equipment and when it is available in the practice, a GP needs to explain why you do or do not use the equipment during a consultation
∎∎∎∎∎	The interpretation of the test result by a GP is the diagnosis, and not the test result itself
∎∎∎∎∎	The usages and results of a test need to be within reasonable time to have value for the GP, otherwise the patient will be referred
∎∎∎∎	Current external actors do not stimulate new use of POC products
∎∎∎∎∎	The GP purchases only a new product when this product is widely validated between actors within the health system
∎∎∎∎∎	When product use is too complex or time consuming for examination and result, the GPs refer the patient to other parties (hospital, diagnostic center)
∎∎∎	Practice owners have a small commercial mind-set for their practice, care first, revenue second
∎∎∎∎∎	POCT results need to be interpreted by the GP to create a diagnosis, and this diagnosis is submitted to the HIS, and therefore, this submission happens manually
∎∎∎∎∎	POC products need to be of a size that can easily fit inside the general practice
∎∎∎	The GP is more cost effective than the secondary care → only 5% of the Dutch care costs are on behalf of the primary care
∎∎∎	The rural areas are more dependent on treatments within the general practice, because of the lack of hospital density within the area

GP: general practitioners. POC: point-of-care, POCT: point-of-care testing.

**Table 4:** Problems relating to the integration of POCT products.

Actor	Actor	Negative Value Transactions	
**GP**			
	GP assistant	∎∎∎∎∎	Complex usages of product, many actions needed to operate
		∎∎∎∎∎	Product usage is time consuming
	POCT	∎∎∎	Many false negatives
		∎∎∎∎∎	Large “grey” diagnosis area of interpretation
	Patient	∎∎∎∎	No decisive answer
